# Frankfurter-Type Sausage Enriched with Buckwheat By-Product as a Source of Bioactive Compounds

**DOI:** 10.3390/foods11050674

**Published:** 2022-02-25

**Authors:** Anna Marietta Salejda, Katarzyna Olender, Magdalena Zielińska-Dawidziak, Monika Mazur, Jakub Szperlik, Joanna Miedzianka, Ireneusz Zawiślak, Joanna Kolniak-Ostek, Aleksandra Szmaja

**Affiliations:** 1 Faculty of Biotechnology and Food Sciences, Wrocław University of Environmental and Life Sciences, 37 Chełmońskiego Str., 51-630 Wrocław, Poland; olendkat@gmail.com (K.O.); monika.mazur@upwr.edu.pl (M.M.); joanna.miedzianka@upwr.edu.pl (J.M.); ireneusz.zawislak@upwr.edu.pl (I.Z.); joanna.kolniak-ostek@upwr.edu.pl (J.K.-O.); aleksandra.szmaja@upwr.edu.pl (A.S.); 2 Faculty of Food Science and Nutrition, University of Life Sciences in Poznań, 48 Mazowiecka Str., 60-623 Poznań, Poland; magdalena.zielinska-dawidziak@up.poznan.pl; 3 Faculty of Biological Sciences, Botanical Garden, University of Wrocław, 23 Sienkiewicza Str., 50-525 Wrocław, Poland; jakub.szperlik@uwr.edu.pl

**Keywords:** agro-industrial by-products, hull, meat products, crop plants, Fagopyrum, meat quality, polyphenols, in vitro protein digestibility, trace elements, amino acids, functional foods

## Abstract

Buckwheat by-products may be used as promising food ingredients due to their nutritional composition. Buckwheat husk (BH) may be used in meat products as a source of valuable compounds. In this study, the addition of BH to the quality of frankfurter-type sausages was investigated, aiming to reduce buckwheat waste and to develop nutritionally enriched sausages. For the purpose of this study, a range of measurements, as well as observations, have been carried out. This included the following: pH, weight losses, yield, the instrumental color and texture measurement, protein digestibility, polyphenols, amino acid, trace elements analysis, and the organoleptic evaluation. Compared with no BH sausages, the cooking losses of sausages with 3% BH were higher, while storage losses were lower. BH increased the hardness of sausages after two weeks of storage. The growing addition of BH resulted in a decrease in L* and b*. This change of color resulted in its lower level of consumer acceptability. BH addition did not reduce the protein digestibility. The total amino acid content increased with the increasing husk addition, from 161.8 mg/kg to 228.0 mg/kg. Moreover, BH increased the content of manganese, calcium, potassium and magnesium. This research suggested that incorporation of BH improved the nutritional value of sausages with minimal changes in technological and sensory properties.

## 1. Introduction

Meat products are generally considered as unhealthy due to the high fat and cholesterol content [1]. For this reason, technologists are trying to expand the meat market by introducing processed products enriched with vegetable ingredients, or with a reduced fat or sodium content. The use of plant products makes it possible to replace the artificial antioxidants with the antioxidants of natural origin, as well as to enrich the product with dietary fiber and n-3 and n-6 fatty acids [2]. Meat, among all available food products, is one of the most susceptible to spoilage. This includes both oxidative changes and microflora development, and subsequent organoleptic changes and the formation of compounds hazardous to the consumer [3,4]. The addition of plant products, rich in natural antioxidants, not only prolongs the shelf life of the product but also has a positive effect on consumer health by making the product a functional one. Studies from numerous authors describe the functional properties of meat products enriched with substances of plant origin. Calvo et al. [5] and Skiepko et al. [6] confirm the anticancer effects of lycopene from tomatoes in dried fermented sausages. In a study by Perales-Jasso et al. [7] oregano oil infused into chorizo showed antioxidant, antimicrobial, antiviral and antifungal activity. The same functional properties have been confirmed for clove oil [8] added to raw pork and for sage oil [9] added to raw and cooked beef, as well as for *Tara spinose* [10,11] or green tea [12,13] added to pork sausages. These ingredients are usually obtained from leftover fruits and vegetable processing, thanks to which they not only enrich the consumer’s diet but also reduce the amount of unused food industry waste. In the groats industry, one of the by-products derived from buckwheat dehulling (*Fagopyrum esculentum* Moench) seeds are hulls (husks). Buckwheat itself contains 30% of dietary fiber, of which about 26.5% is an insoluble fiber fraction, essential amino acids, polyunsaturated fatty acids and vitamins B and E [14]. Due to the high fiber content, the addition of buckwheat to food products slows down their digestion, so that the feeling of satiety after a meal remains longer [15]. Apart from its following functional properties, such as being antioxidant, anti-carcinogenic and anti-inflammatory, buckwheat also contains a high content of flavonoids, as well as rutin, quercetin, orientin, vitexin, isovitexin and isoorientin [16,17]. Buckwheat effectively limits weight gain, also causing a decrease in the content of blood cholesterol, especially LDL fraction [18]. Buckwheat is rich in potassium, boron, iron, copper, chromium, zinc, cobalt, nickel, calcium, magnesium, phosphorus and sodium [19,20]. The content of these elements varies depending on its degree of processing [21,22]. Husk, as a by-product of buckwheat processing, is also characterized by the high content of mineral compounds (P, K, Mg, Mn, Zn, Cu), vitamins of group B and E, as well as dietary fiber, which is why they are usually used for the production of high-fiber preparations. It is a source of tannins and phenolic compounds with antioxidant properties, including rutin and quercetin. Due to its chemical composition, buckwheat husk has a beneficial effect on the human body; among others, it supports immunity, has anti-oxidative, -microbial, -inflammatory and -rheumatic properties [17,23,24,25]. This by-product is utilized in the production of mattress fillings and pillows [26,27] and as a component of biodegradable packaging [28]. Ash obtained from buckwheat husk can replace sawdust in the manufacture of easily fusible clay bodies [29]. This raw material can also be used in the production of pellets and fuel briquettes or alternative fuel (biomethanol) [26,30]. It is also used in herbal medicine and the pharmaceutical industry, as an ingredient in teas and dietary supplements [26]. However, to the best of our knowledge, buckwheat husk use in the food industry is very limited. The studies of Wronkowska et al. [31], demonstrated that commercially available bakery products can be enriched with raw and roasted buckwheat husk, due to their positive impact on sensory characteristics, consumer acceptance and microbiological properties of bread/rolls after storage. Hęś et al. [32] proved the positive effect of buckwheat hull water extract on lipid oxidation in frozen-stored meatballs from ground pork. The extract showed stronger antioxidant activity, higher ability to scavenge free radicals and bind iron ions than synthetic antioxidants (BHT), therefore, justifying the application of this additive. However, obtaining the additive used was a time- and cost-intensive process, so in the presented study we decided to find a cheaper solution by introducing only ground husks to the formulation of frankfurter-type sausages. Therefore, the objective of the study was to obtain a new generation of sausages, enriched with buckwheat husk, which is a source of bioactive compounds. It was also evaluated how the buckwheat husk would affect the technological and sensory quality of frankfurter-type sausages.

## 2. Materials and Methods

### 2.1. Preparation of Sausages Samples with Buckwheat Husk

The research material consisted of frankfurter-type sausages differing in the proportion of buckwheat husk (BH) in the formulation (Table 1). For this purpose, ham cuts (*M. semimembranosus*) and backfat were obtained from Dworecki Meat Processing Plant (Golejewo, Poland) 48 h *post-mortem*. Raw materials were passed through a meat grinder (Zelmer, fi 6), then 200 g of meat and 120 g of fat were mixed with ice, curing salt (4.8 g, Żuk-Pol, Wrocław, Poland), sugar, seasoning (Bellako sp. z o.o., Zabrze, Poland) and the appropriate amount of ground buckwheat husk (0% BH = 0 g, 1% BH = 4 g, 2% BH = 8 g and 3% BH = 12 g, respectively, Młyn Niedźwiady, Kalisz, Poland). The mixture was homogenized for 3 sec at 9000 rpm with a Büchi Mixer B400 (BÜCHI Labortechnik GmbH, Flawil, Germany). The homogenized mass was weighed (approximately 60 g) in polypropylene tubes (2.5 × 12 cm) and then heat treated in a water bath (99 °C, Julabo TW12, Julabo Inc., Allentown, PA, USA) until a temperature of 72 °C was reached at the geometric center of sample. Once this temperature was reached, the product was cooled on ice. The cooled products were vacuum packed in multilayer PA/PE bags and stored at 4 ± 2 °C for further analyses. The sausages were manufactured in two production batches on different days.

### 2.2. pH Value

The negative logarithm of the hydrogen ion concentration in the sausages was determined using a pH meter (Orion 3-Star pH Benchtop Meter, Thermo Fisher Scientific, Waltham, MA, USA). Two measurements were made directly in the sausage samples after their preparation and after reaching ambient temperature.

### 2.3. Weight Losses and Yield

The weight losses measured after thermal treatment and after two weeks of cold storage were expressed as a percentage of initial sample weight. Yield of the production process was determined as the proportion of the weight after (weight of cooked meat stuffing) and weight before thermal treatment (weight of uncooked meat stuffing).

### 2.4. Instrumental Texture Profile Analysis

For texture profile analysis (TPA), a Zwick/Roell Z010 testing machine (Zwick Testing Machines Ltd., Leominster, Herefordshire, UK) was used. The analysis was performed on samples of cylindrical shape (15 × 27 mm, H × d). Next, 75% compression of samples was performed between two parallel plates; each cycle was performed at the same head speed (60 mm/min) and relaxation time of 30 s. Texture parameters such as hardness [N], cohesiveness, elasticity [mm], chewiness [Nm] and gumminess were evaluated at room temperature (22 ± 1 °C), directly after the production process and after 2 weeks of cold storage.

### 2.5. Instrumental Color Measurement

The instrumental color measurement of sausages was performed on the day of production and after two weeks of cold storage. Sausages were cut crosswise into 10–15 mm thick slices. Color of the surface was measured using a Konica Minolta Chroma Meters CR-400 (Osaka, Japan) and was expressed by L (lightness), a (redness), and b (yellowness) parameters in CIE Lab system. The chroma meter was set at D65 illuminant and 10° standard observer and calibrated before each measurement against a standard white tile (*Y*  =  93.8; *x*  =  0.313; *y*  =  0.319).

### 2.6. Organoleptic Evaluation 

The organoleptic evaluation was conducted on the day of the production for each production variant. All products were evaluated by the same team of evaluators, consisting of 16 persons aged 21–23 years in a 50/50 gender distribution. The evaluation was aimed at investigating the acceptability of selected sensory attributes of frankfurter-type sausages enriched with BH. The following attributes were evaluated: color, taste, smell, texture and firmness. Samples of cylindrical shape (15 × 27 mm, H × d) marked with 4-character code were subjected to the evaluation at room temperature (22 ± 1 °C), under white light. The evaluation was carried out by the scaling method according to a 9-point acceptance scale, where 1 meant “extremely dislike” and 9 meant “extremely like”. A ranking assessment in which evaluators were asked to rank the samples from least to most preferred was also conducted.

### 2.7. Identification of Polyphenols 

The extraction of polyphenols was determined using the method described by Püssa et al. [33] and previously described by Mazur et al. [34] with its own modifications. The samples of homogenized sausages (0.5 ± 0.01 g) were extracted with a solution, having a pH of 2.58, with 5 mL of methanol (80%) and HCl (0.1%). Next, the samples were shaken for 30 min at room temperature and centrifuged (5000 rpm, 10 min). After the addition of hexane (10 mL) to the supernatant, the hydrophilic layer was obtained and kept in Eppendorf vials at 18 °C until analysis. Extractions were carried out in duplicate. Before the identification the sample was diluted with 2% formic acid (1:1 *v*/*v*, Sigma-Aldrich, Steinheim, Germany). For the identification of polyphenols, the protocol proposed by Kolniak-Ostek [35] was used. The identification of polyphenols in sausage extracts was carried out using an ACQUITY Ultra Performance LC system equipped with a photodiode array detector with a binary solvent manager (Waters Corporation, Milford, MA, USA) with a mass detector G2 Q-Tof micromass spectrometer (Waters, Manchester, UK) equipped with an electrospray ionization (ESI) source operating in negative mode. For the separation of single polyphenols, an UPLC BEH C18 column (1.7 µm, 2.1 × 100 mm, Waters) was used. The mobile phase consisted of 0.1% formic acid, *v*/*v* (solvent A) and 100% acetonitrile (solvent B). The single components of polyphenols were characterized based on the retention time and the accurate molecular masses. The data obtained from UPLC-MS were analyzed in the MassLynx 4.0 ChromaLynx (Application Manager software, Waters). Phenolic acids were monitored at 320 nm, flavanols at 280 nm, flavonols at 360 nm and flavones at 340. The PDA spectra were measured over the wavelength range of 200–600 nm in steps of 2 nm. The retention times and spectra were compared to those of the authentic standards (chlorogenic acid, quinic acid and apigenin di-glucoside were purchased from Extrasynthese, Lyon, France).

### 2.8. Amino Acid Analysis 

The amino acid composition of samples was determined by ion-exchange chromatography after 23 h hydrolysis with 6 N HCl at 110 °C. After cooling, filtering and washing, the hydrolyzed sample was evaporated in a vacuum evaporator at a temperature below 50 °C. The dry residue was dissolved in a buffer of pH 2.2. The prepared sample was analyzed using the ninhydrin method [36,37]. The pH 2.6, 3.0, 4.25, and 7.9 buffers were applied. The ninhydrin solution was buffered at pH 5.5. The hydrolyzed amino acids were determined using an AAA-400 analyzer (INGOS, Prague, Czech Republic). A photometric detector was used, working at two wavelengths, 440 nm and 570 nm. A column of 350 × 3.7 mm, packed with ion exchanger Ostion LG ANB (INGOS) was utilized. Column temperature was kept at 60–74 °C and detector at 121 °C. The calculations were carried out according to an external standard. No analysis of tryptophan was carried out. 

### 2.9. In Vitro Protein Digestibility Determination

Samples were digested with the in vitro method simulating multienzymatic two-stage (gastric and intestinal) digestion [38,39]. The oral stage was omitted as irrelevant to protein as well as the large intestine digestion. As such, 1.5 g of the studied samples were introduced into distilled water containing pepsin (60,000 U) (Sigma, St. Louis, MO, USA). Then pH of the mixture was lowered to 2.0 by addition of 1 M HCl. The first stage of digestion (gastric) was carried out at 37 °C, for 2 h, with shaking. Then, the pH of the mixture was increased to 7.4 (with 1 M NaHCO_3_) and a solution containing pancreatic–intestine extract (0.005 g, Sigma, St. Louis, MO, USA) and bile salts (0.03 g, Sigma, St. Louis, MO, USA) was introduced to simulate intestine digestion. This stage was continued for 2 h at 37 °C with shaking. After digestion samples were centrifuged. Non-digested proteins present in the supernatant were precipitated with 10% TCA (trichloroacetic acid). The percentage content of protein nitrogen released into the obtained supernatants was compared to the content of protein in the non-digested samples with the use of the Kjeldahl method [40].

### 2.10. Determination of Trace Elements

The samples were mineralized “wet” in a closed microwave system. Following this, 5 cm^3^ of concentrated nitric acid (V) (CTC—reagent grade) was added to an aliquot of a homogeneous sample (from 0.1 g to 0.5 g), then the samples were mineralized in the MARS 6 microwave samples preparation system. The mineralisates were quantitatively transferred to 10 cm^3^ measuring vessels with redistilled water. The mineralization was carried out in accordance with PN-EN 13805:2003 [41]. Atomic emission spectrometry using the SpectraAA atomic absorption spectrometer with the flame attachment of AA240FS (VarianTechtron Pty Ltd., Mulgrave, Australia) for the determination of the trace elements was used. The determination of the content of calcium, sodium and potassium was carried out in accordance with PN-EN 1134:1999 [42]. While the determination of the content of zinc, iron, magnesium, manganese and copper was carried out in accordance with PN-EN 14084:2004 [43]. The methods were validated using BCR-185R certified reference material-bovine liver, the measurement uncertainty was estimated at 5%.

### 2.11. Statistical Analysis

The collected data of the two production batches were analyzed statistically with TIBCO Statistica, version 13.3 (TIBCO Software Inc., Palo Alto, CA, USA). Two-factor analysis of variance (ANOVA) and Duncan’s multiple range test allowed for the determination of the statistically significant differences. Results are presented as mean ± SE (standard error). 

## 3. Results and Discussion

The applied additive in the form of ground buckwheat husk (BH) did not affect the pH value of sausages, which was within the range 6.0–6.2. However, the addition of BH had a significant effect on the weight losses during the thermal treatment and at the same time, on the process yield (Table 2).

The experimental products lost between 14% and 15.5% of their initial weight and the yield of production ranged from 84.5 to 85.8%. The highest weight losses during cooking and the lowest values of yield were observed in sausages with 3% addition of buckwheat husk. On the other hand, during storage, BH effectively reduced weight losses from 1.93% in the control sausages to 1.36% in sausages with its highest content. This indicates that the addition of buckwheat husk has a positive effect on water retention during the storage of frankfurter-type sausages. These results are supported by the study of Sol-Hee Lee et al. [44], in which a homogenized sausage-type product retained more moisture along with the increasing buckwheat powder addition. Buckwheat constitutes a rich source of fiber, enhancing the water-holding capacity and protein-binding ability of the meat [45]. Many authors have confirmed the positive effect of added fiber sources, e.g., soy [46], sugarcane [47], oat [48], pumpkin [49], wheat and carrot [50], on water retention in meat products.

In our research, the share of buckwheat husk in the composition also resulted in the changes in the texture profile of sausages (Table 3). At the day of the production, the sausages containing buckwheat husk, at the 1 and 2% levels, were double compressed with less force than the sausages with 3% BH and the control sample. After two weeks of cold storage, this relationship changes and in the control sample, the force required for deformation did not change, while in all the sausages with the buckwheat husk addition, the force increased and the products presented higher hardness. The springiness on the day of production was the same for all products and increased during two weeks of cold storage. Similar changes were observed in the measurement of chewiness and cohesion. Immediately after the production, the lowest gumminess was measured in samples with 1% BH, values of this parameter obtained in the variants of 2% and 3% of BH addition did not differ from those in control samples. However, after storage time, gumminess values increased in all variants. The addition of buckwheat husk changes the texture profile mainly after two weeks of storage. These products were more compact and firmer than the sausages produced without buckwheat husk in the formulation. The increase in hardness might be explained by the ability of plant fiber to form a stronger three-dimensional network within the meat matrix [51]. Similar results were obtained by Bejosano and Corke [52] in their study on the effect of buckwheat on homogenized meat products, where buckwheat proteins were a good substitute for meat in product formulation, behaved similarly to soy proteins and increased the firmness of the product. 

Table 4 shows the results of color measurement in samples immediately after production and after two weeks of cold storage.

The variants differed significantly in the values obtained for the measurement of the L* parameter, directly after the production, i.e., along with the increase in the share of buckwheat husk, the sausages were darker. The same results were obtained by Salejda et al. [53], by adding the by-product of walnut processing to homogenized sausages, Kim et al. [54], by the addition of germinated barley to cooked chicken sausages, Shin et al. [55], by adding black garlic extract to sausages that were stored for 4 weeks, as well as Seo et al. [56], by adding *Caesalpinia sappan* L. (CS) extract to cooked pork sausages. In our own research, the growing addition of buckwheat husk also resulted in the decrease in yellowness of frankfurter-type sausages. Saturation of b* also decreased during cold storage. The observed changes in color are probably related to the color of the BH (L* 45.2, a* 4.65, b* 9.94). 

**Figure 1:**
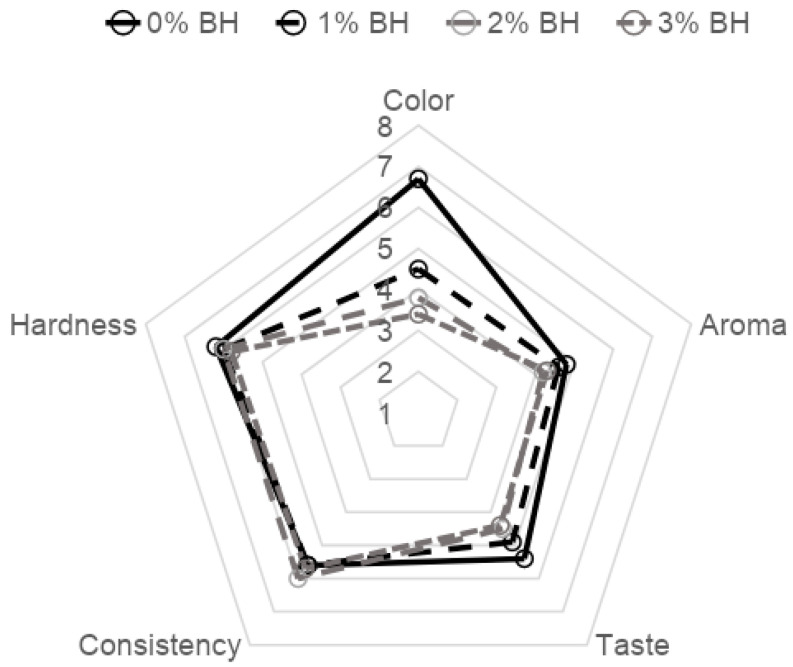
Results of organoleptic evaluation.

In both production batches, the acceptability of color, aroma, taste, consistency and hardness of frankfurter-type sausages were evaluated. The average results of these evaluations are summarized in Figure 1. Buckwheat husk addition caused a color change (darkening) in sausages, which was confirmed by the instrumental measurement of this parameter. This change resulted in lower acceptability of this parameter by the evaluation team. The best score (6.7) was received for samples of sausages manufactured without buckwheat husk. In the case of sausages with buckwheat husk, products with the lowest addition of BH were rated highest. Therefore, as it was suggested by Jin et al. [57], it is worth thinking about discoloration or bleaching of the BH before its application to sausages on an industrial scale. The evaluators also observed differences in aroma between products, giving the highest mark to the control sausages and the lowest to the samples with 2% of buckwheat husk addition (the difference between them was 0.59 points). Significantly, samples with and without the 1% additive were evaluated similarly (4.8 and 4.6 points, respectively). Increasing the concentration of BH in the composition resulted in a decrease in taste acceptability, and as in the evaluation of aroma, the differences between the control sample and the sample with 1% of BH were not significant. The consistency was slightly better in products with buckwheat husk added than in the control sample, which was not confirmed in the assessment of the last evaluated characteristic (hardness). The evaluation team also ranked the sausages in order from least to most preferred. The control sample appeared to be the most preferred product, although the difference in the number of indications between variants was very small, i.e., between the variant 0% BH and 1% BH, there was only one point, and between 0% BH and variants 2% BH and 3% BH, only two points. 

**Figure 2:**
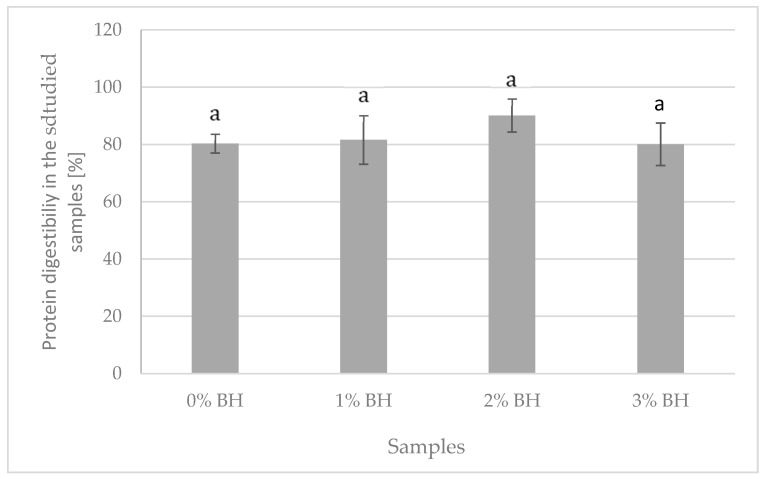
Effect of BH on protein digestibility in the studied samples. a—same letters indicate no significant differences (*p* ≤ 0.05) between values.

As shown in Table 5, a significant effect of the BH addition on the amino acids content in sausages was observed. The total amino acid content increased with increasing husk addition. The highest content (228.0 mg/kg) was recorded in sausages with 3% BH and the lowest (161.8 mg/kg) in sausages manufactured without its addition. This more than 40% difference in content is due to the well-balanced composition of amino acids in buckwheat, including its derivatives [58]. Sausages with buckwheat husk were characterized by significantly higher content of qualitatively determined amino acids. Especially sausages with 2 and 3% of buckwheat husk addition, containing over 40% more of most of the assayed amino acids than the samples without the addition. They also contained half as much threonine, cysteine, isoleucine and phenylalanine and almost twice as much alanine and tyrosine. As in the works of other authors [59,60], the observed increase in the content of amino acids results from the fact that, in control samples, their content determines only animal protein, while in samples with a modified recipe, this content determines the presence of both animal and plant protein. Importantly, the used additive did not reduce the digestibility of protein in the studied samples (Figure 2). 

Decreased digestibility of foodstuffs produced with the addition of husk [15,61,62] results from increased content of antinutrients present in this part of seeds, i.e., high content of tannin, fiber, polyphenol, inhibitors etc. [62]. This is a desirable effect for starchy (but not high-protein) products, such as sausages. Addition up to 3% of buckwheat husk, which itself is also a source of protein, does not modify the protein bioavailability from the studied material, despite the fact that it brings the abovementioned amounts of dietary fiber (mainly insoluble) and flavonoids into the product. Prior studies confirmed decreased lipid and starch digestibility of varied foodstuffs, with the increased content of fiber [63,64]. However, the addition of insoluble fiber, up to 4%, did not significantly influence protein digestibility [39]. Moreover, it was observed that phenolic compound content may not affect protein digestibility [65].

**Figure 3:**
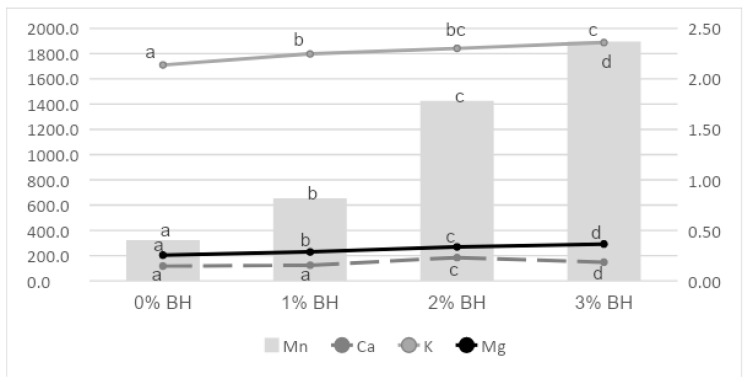
Effect of BH on mineral content in sausages (mg/kg). a–d—different letters indicate significant differences (*p* ≤ 0.05) between values.

Processing buckwheat husk into frankfurter-type sausages had a significant effect on mineral content (Figure 3). The addition of this non-meat ingredient increased the content of manganese, calcium, potassium and magnesium, whereby the first of these trace elements increased almost six times, while the last, by over 40 percent. These minerals are valuable for dietary and technological reasons [66]. A significant increase in magnesium and manganese, after the addition of a non-meat ingredient to a meat product, also confirmed the studies of Zając et al. [67], on the quality of pork loaves with the addition of hemp seeds, de-hulled hemp seeds, hemp protein and hemp flour. The replacement of lean meat/fat portion with house cricket flour also fortifies manganese, potassium and magnesium content of meat emulsion [68].

Sausages based on buckwheat husk can also be an excellent source of phenolic compounds. We found the following components in the studied sausages (Table 6): (+)-catehin or (−)-epicatechin, quercetin-3-(6-malonyl)-glucoside, trehalose dihydrate, inosine-5′-monophosphate, 7-hydroxy-4′-methoxyisoflavone (formononetin), sinapic acid, juniperic acid, isorhamnetin-3-galactoside-6″-rhamnoside, kaempferol-7-*O*-alpha-l-rhamnoside, sinapinaldehyde, juniperic acid, isorhamnetin-3-galactoside-6″-rhamnoside, kaempferol-7-*O*-alpha-l-rhamnoside, sinapinaldehyde, quercetin derivatives, rosmarinic acid, flavonoid derivatives, vitexin (apigenin-8-C-glucoside), apigenin-7-*O*-glucoside, apigenin, kaempferol-3-glucoside-3″-rhamnoside, vitexin-2″-*O*-rhamnoside.

**Figure 4:**
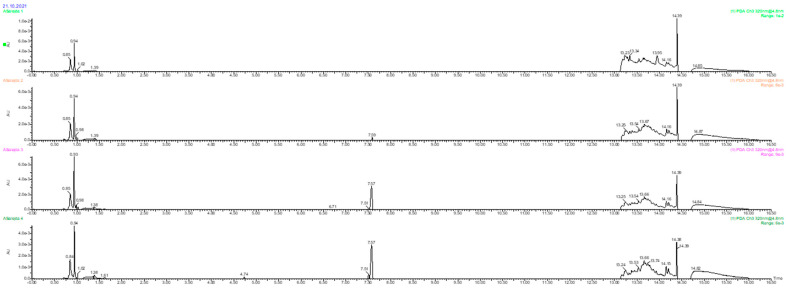
Comparison of representative UPLC-MS chromatogram of studied sausages.

The main phenolic compounds identified in the studied products were vitexin and their derivatives, except in the control. The variant with the highest addition of buckwheat husk was found to contain the highest concentration of those compounds (Figure 4: Rt 7.57; 7.59). The second most abundant compound was quercetin, also a flavonoid. It is in agreement with Wahlsten [69], who also identified vitexin and quercetin derivatives in *Fagopyrum esculentum*. Both, quercetin and vitexin were characterized by antioxidant activity stronger than synthetic antioxidant BHT [70]. These flavonoids had significant anti-inflammatory effects and, according to Nikfarjam et al. [71], may be considered as a therapeutic strategy for treating patients with neutrophil-mediated inflammatory diseases. Vitexin and quercetrin, isolated from *Serjania erecta* Radlk leaves, protect PC12 cells from Aβ_25–35_ peptide-induced toxicity [72]. 

## 4. Conclusions

We concluded that the buckwheat husk can be used as a non-meat additive to increase the nutritional value of frankfurter-type sausages, without compromising their technological quality. Notably, BH sausages appeared to be a good dietary source of essential amino acids, trace elements and phenolic compounds. Further studies should take into account the impact of BH on the microbial quality of sausages, as well as in vivo testing. These studies will allow for the application of BH as a commercial food additive to develop novel functional sausages. The potential consumer should be aware of the beneficial effects of these new products on health and be convinced to consume them, despite differences in color and taste, or consider the modification of recipe or production technology limiting this adverse effect.

## Figures and Tables

**Table 1:** Composition of sausages enriched with BH [g].

Ingredients [g]	Formulation			
	0% BH	1% BH	2% BH	3% BH
Pork	200	200	200	200
Back fat	120	120	120	120
Ice	80	80	80	80
Curing salt	4.8	4.8	4.8	4.8
Sugar	4	4	4	4
Seasoning	2.4	2.4	2.4	2.4
Buckwheat husk (BH)	0	4	8	12

**Table 2:** Effect of BH on thermal and storage losses and yield of production (%).

Formulation	Thermal Losses	Storage Losses	Yield of Production
0% BH	14.0 a ± 0.53	1.93 d ± 0.01	85.8 b ± 0.38
1% BH	14.2 a ± 0.67	1.69 c ± 0.01	85.8 b ± 0.74
2% BH	15.5 c ± 0.53	1.52 b ± 0.01	84.5 a ± 0.53
3% BH	14.9 bc ± 0.52	1.36 a ± 0.01	85.5 b ± 0.89

a, b, c, d—different letters indicate significant differences (*p* ≤ 0.05) between values.

**Table 3:** Effect of BH on texture parameters of sausages.

Storage Time [Weeks]	Formulation	Hardness [N]	Springiness [mm]	Gumminess [N]	Chewiness [Nm]	Cohesiveness [–]
0	0% BH	52.9 bc ± 1.76	0.68 a ± 0.06	18.2 b ± 1.02	12.2 a ± 1.71	0.33 a ± 0.03
	1% BH	47.6 a ± 1.33	0.68 a ± 0.06	14.8 a ± 1.20	10.1 a ± 1.55	0.31 a ± 0.02
	2% BH	49.4 ab ± 4.31	0.65 a ± 0.03	17.3 ab ± 1.34	11.4 a ± 0.82	0.34 ab ± 0.04
	3% BH	52.1 bc ± 1.94	0.68 ab ± 0.03	16.9 ab ± 1.78	11.5 a ± 1.02	0.33 a ± 0.04
2	0% BH	54.3 cd ± 3.58	0.70 bc ± 0.05	22.7 c ± 4.31	16.0 b ± 4.00	0.42 d ± 0.04
	1% BH	60.0 d ± 2.66	0.75 c ± 0.03	25.6 d ± 1.96	19.1 c ± 2.04	0.43 d ± 0.03
	2% BH	58.7 d ± 2.16	0.75 c ± 0.04	23.6 cd ± 3.96	17.3 cb ± 1.68	0.38 c ± 0.03
	3% BH	58.0 d ± 1.67	0.73 bc ± 0.04	21.2 c ± 1.93	15.4 c ± 1.04	0.37 bc ± 0.04

a, b, c, d—different letters indicate significant differences (*p* ≤ 0.05) between values.

**Table 4:** Effect of BH on color parameters of sausages.

Storage Time [Weeks]	Formulation	L*	a*	b*
0	0% BH	74.21 d ± 0.99	4.55 b ± 0.47	10.40 c ± 0.35
	1% BH	62.80 c ± 0.92	4.10 a ± 0.22	7.55 b ± 0.36
	2% BH	57.56 b ± 2.51	4.42 b ± 0.35	7.04 b ± 0.26
	3% BH	53.64 a ± 1.39	4.60 b ± 0.25	6.98 a ± 0.47
2	0% BH	74.55 d ± 0.54	5.98 c ±0.70	8.89 c ± 0.36
	1% BH	64.67 c ± 1.54	3.66 a ± 0.16	6.96 b ± 0.33
	2% BH	58.21 b ± 1.55	3.38 a ± 0.29	6.61 b ± 0.30
	3% BH	52.87 a ± 1.88	4.94 b ± 0.66	5.88 a ± 0.47

a–d—different letters indicate significant differences (*p* ≤ 0.05) between values.

**Table 5:** Effect of BH on amino acid content in sausages (g/mg).

Amino Acid	0% BH	1% BH	2% BH	3% BH
ASP	16.4 a ± 0.05	20.7 b ± 0.31	21.0 b ± 0.01	21.5 b ± 0.39
THR	7.92 a ± 0.02	10.0 b ± 0.18	11.9 c ± 0.00	12.0 c ± 0.02
SER	6.52 a ± 0.01	8.19 b ± 0.15	10.6 c ± 0.01	10.9 d ± 0.02
GLU	29.6 a ± 0.14	38.5 c ± 0.54	32.8 d ± 0.01	40.4 c ± 0.22
PRO	6.29 a ± 0.10	5.81 a ± 0.06	9.25 c ± 0.15	7.90 b ± 0.35
GLY	10.3 a ± 0.04	11.5 b ± 0.15	13.1 d ± 0.01	12.7 c ± 0.03
ALA	10.0 a ± 0.00	12.2 b ± 0.11	18.1 d ± 0.00	16.1 c ± 0.01
CYS	0.37 a ± 0.01	0.43 a ± 0.05	0.43 a ± 0.01	0.56 b ± 0.00
VAL	8.65 a ± 0.03	11.1 b ± 0.19	12.8 c ± 0.07	11.1 b ± 0.06
MET	3.03 a ± 0.01	3.58 b ± 0.05	4.41 d ± 0.00	4.17 c ± 0.01
ILE	7.94 a ± 0.00	10.2 b ± 0.17	12.1 c ± 0.02	12.1 c ± 0.03
LEU	13.4 a ± 0.04	17.1 b ± 0.09	16.8 b ± 0.29	17.1 b ± 0.11
TYR	3.71 a ± 0.00	5.06 b ± 0.07	6.04 c ± 0.00	7.38 d ± 0.03
PHE	7.28 a ± 0.00	9.20 b ± 0.16	11.5 d ± 0.01	10.9 c ± 0.05
HIS	5.42 a ± 0.03	7.39 b ± 0.14	5.73 a ± 0.01	7.73 c ± 0.07
LYS	13.8 a ± 0.01	17.8 b ± 0.33	18.3 b ± 0.10	19.8 c ± 0.04
ARG	11.2 a ± 0.04	14.0 b ± 0.15	15.4 c ± 0.00	15.8 d ± 0.05
Total	161.8 a	202.7 b	220.2 c	228.0 d

a–d—different letters indicate significant differences (*p* ≤ 0.05) between values.

**Table 6:** The phenolic compounds identified in sausages with buckwheat husk addition.

PDA RT (min)	TOF1	TOF2	[M − H]^−^ (*m/z)*	Fragment Ions	Compound
0.85	0.86	0.88	273.0530		Either catehin or epicatechin
0.94	1.051	1.035	-	146.1843; 188.1502	Quercetin-3-(6-malonyl)-Glucoside
	1.018	1.035	377.1273		Trehalose dihydrate
0.98	1.604	1.567	-	347.0975	Inosine-5′-monophosphate
1.02	2.447	2.430	267.1691		7-Hydroxy-4′-Methoxyisoflavone (Formononetin)
	3.188	3.171	164.2030		Sinapic acid
	3.532	3.527	271.1116		Juniperic acid
	5.325	5.317	315.1732		Isorhamnetin-3-Galactoside-6″-Rhamnoside
	5.581	5.560	-	257.0191	Kaempferol-7-*O*-alpha-l-rhamnoside
	5.857	5.874	207.0349		Sinapinaldehyde
	5.945	5.962	-	433.9072	Quercetin derivatives
	6.255	6.300	-	160.9744; 197.9301	Rosmarinic acid
	6.821	6.852	415.1234	253.1580	Flavonoid derivatives
7.59	7.718	7.789	431.1103	311.1306	Vitexin Apigenin-8-C-glucoside
	8.183	8.200	431.1018	269.1367	Apigenin-7-*O*-glucoside
	10.474	10.522	-	253.1515	Flavonoid derivatives
	10.697	10.714	269.1435	-	Apigenin
	13.265	13.314	595.1830		Kaempferol-3-Glucoside-3″-Rhamnoside
	14.471	14.522	-	311.2462; 293.2622	Vitexin-2″-*O*-rhamnoside
	14.539	14.589	-	253. 259. 265	Unidentified flavonoid

## Data Availability

Data sharing is not applicable. No new data were created or analyzed in this study.

## References

[1] Serrano A., Librelotto J., Cofrades S., Sánchez-Muniz F.J., Jiménez-Colmenero F. (2007). Composition and physicochemical characteristics of restructured beef steaks containing walnuts as affected by cooking method. Meat Sci..

[2] Hygreeva D., Pandey M.C., Radhakrishna K. (2014). Potential applications of plant based derivatives as fat replacers, antioxidants and antimicrobials in fresh and processed meat products. Meat Sci..

[3] Kanner J. (1994). Oxidative processes in meat and meat products: Quality implications. Meat Sci..

[4] Fung D.Y. (2010). Microbial hazards in food: Food-borne infections and intoxications. Handbook of Meat Processing.

[5] Calvo M.M., García M.L., Selgas M.D. (2008). Dry fermented sausages enriched with lycopene from tomato peel. Meat Sci..

[6] Skiepko N., Chwastowska-Siwiecka I., Kondratowicz J. (2015). Properties of lycopene and utilizing it to produce functional foods. Zywnosc-Nauka Technol. Jakosc.

[7] Perales-Jasso Y.J., Gamez-Noyola S.A., Aranda-Ruiz J., Hernandez-Martinez C.A., Gutierrez-Soto G., Luna-Maldonado A.I., Silva-Vazquez R., Hume M.E., Mendez-Zamora G. (2018). Oregano powder substitution and shelf life in pork chorizo using Mexican oregano essential oil. Food Sci. Nutr..

[8] Shan B., Cai Y., Brooks J.D., Corke H. (2009). Antibacterial and antioxidant effects of five spice and herb extracts as natural preservatives of raw pork. J. Sci. Food Agric..

[9] Fasseas M.K., Mountzouris K.C., Tarantilis P.A., Polissiou M., Zervas G. (2008). Antioxidant activity in meat treated with oregano and sage essential oils. Food Chem..

[10] De la Cruz-Lapa P. (2004). An integral and rational utility of tara (*Caesalpinia spinosa*). Rev. Inst. Investig. FIGMMG.

[11] Skowyra M., Janiewicz U., Salejda A.M., Krasnowska G., Almajano M.P. (2015). Effect of Tara (*Caesalpinia spinosa*) Pod Powder on the Oxidation and Colour Stability of Pork Meat Batter During Chilled Storage. Food Technol. Biotechnol..

[12] Weisburger J.H., Veliath E., Larios E., Pittman B., Zang E., Hara Y. (2002). Tea polyphenols inhibit the formation of mutagens during the cooking of meat. Mutat. Res. Genet. Toxicol. Environ. Mutagenesis.

[13] Salejda A.M., Krasnowska G., Tril U. (2011). Attempt to utilize antioxidant properties of green tea extract in the production of model meat products. Zywnosc-Nauka Technol. Jakosc.

[14] Lee M., Woo S., Oh S., Kwon T. (1995). Changes in contents and composition of insoluble dietary fiber during buckwheat germination. Korean J. Food Nutr..

[15] Gordon D. (1992). The importance of dietary fiber in human nutrition and health. Korean J. Nutr..

[16] Baumgertel A., Grimm R., Eisenbeiss W., Kreis W. (2003). Purification and characterization of a flavonol 3-O-betaheterodisaccharidase from the dried herb of Fagopyrum esculentum. Phytochemistry.

[17] Dziedzic K., Górecka D., Kobus-Cisowska J., Jeszka M. (2010). Możliwość wykorzystania gryki w produkcji żywności funkcjonalnej. Nauka Przyr. Technol..

[18] Choy A.L., Morrison D.P., Hughes G.J., Marriott J.P., Small M.D. (2013). Quality and antioxidant properties of instant noodles enhanced with common buckwheat flour. J. Cereal Sci..

[19] Trzebska-Jeske I., Nadolna I., Rutkowska U., Secomska B. (1973). Wpływ obróbki mechanicznej na wartość odżywczą kasz produkowanych w kraju. Rocznik PZH.

[20] Zalesskaja E.W., Mielnikow E.M. (1978). Izmienije mineralnogo sostawa jadricy pri gidrotermiczeskoj obrabotke. Technology.

[21] Amarowicz R., Fornal Ł. (1987). Characteristics of buckwheat grain mineral components and dietary fiber. Fagopyrum.

[22] Ikedai S., Yamashita Y., Kreft I. (1999). Mineral composition of buckwheat by-products and its processing characteristics to konjak preparation. Fagopyrum.

[23] Sedej I., Sakac M., Mandic A., Misan A., Tumbas V., Canadanovic-Brunet J. (2012). Buckwheat (*Fagopyrum esculentum* Moench) grain and fractions: Antioxidant compounds and activities. J. Food Sci..

[24] Hęś M., Gorecka D., Dziedzic K. (2012). Antioxidant properties of extracts from buckwheat by-products. Acta Sci. Pol. Technol. Aliment..

[25] Dziadek K., Kopeć A., Pastucha E., Piątkowska E., Leszczyńska T., Pisulewska E., Witkowicz R., Francik R. (2016). Basic chemical composition and bioactive compounds content in selected cultivars of buckwheat whole seeds, dehulled seeds and hulls. J. Cereal Sci..

[26] Borkowska B., Robaszewska A. (2012). Use of buckwheat grain in various industry branches. Zesz. Nauk. Akad. Mor. Gdyni.

[27] Zarzecka K., Gugała M., Mystkowska I. (2014). Wartość odżywcza i możliwości wykorzystania gryki. Postępy Fitoter..

[28] Ecovative. https://ecovativedesign.com/mycocomposite.

[29] Siauciunas R., Valanciene V. (2020). Influence of buckwheat hulls on the mineral composition and strength development of easily fusible clay body. Appl. Clay Sci..

[30] Cucina M., Pezzolla D., Tacconi C., Gigliotti G. (2021). Pretreatments for enhanced biomethane production from buckwheat hull: Effects on organic matter degradation and process sustainability. J. Environ. Manag..

[31] Wronkowska M., Zieliński H., Szmatowicz B., Ostaszyk A., Lamparski G., Majkowska A. (2019). Effect of roasted buckwheat flour and hull enrichment on the sensory qualities, acceptance and safety of innovative mixed rye/wheat and wheat bakery products. J. Food Process. Preserv..

[32] Hęś M., Szwengiel A., Dziedzic K., Le Thanh-Blicharz J., Kmiecik D., Górecka D. (2017). The Effect of Buckwheat Hull Extract on Lipid Oxidation in Frozen-Stored Meat Products. J. Food Sci..

[33] Püssa T., Pällin R., Raudsepp P., Soidla R., Rei M. (2008). Inhibition of lipid oxidation and dynamics of polyphenol content in me-chanically deboned meat supplemented with sea buckthorn (*Hippophae rhamnoides*) berry residues. Food Chem..

[34] Mazur M., Salejda A.M., Pilarska K.M., Krasnowska G., Nawirska-Olszańska A., Kolniak-Ostek J., Bąbelewski P. (2021). The Influence of Viburnum opulus Fruits Addition on Some Quality Properties of Homogenized Meat Products. Appl. Sci..

[35] Kolniak-Ostek J. (2016). Chemical composition and antioxidant capacity of different anatomical parts of pear (*Pyrus communis* L.). Food Chem..

[36] Simpson R.J., Neuberger M.R., Lin T.Y. (1976). Complete amino acid analysis of proteins from a single hydrolysate. J. Biol. Chem..

[37] Spackman D.H., Stein W.H., Moore S. (1958). Automatic recording apparatus for use in the chromatography amino acid. Anal. Chem..

[38] Kowalczewski P.Ł., Olejnik A., Białas W., Rybicka I., Zielińska-Dawidziak M., Siger A., Kubiak P., Lewandowicz G. (2019). The nutritional value and biological activity of concentrated protein fraction of potato juice. Nutrients.

[39] Zielińska-Dawidziak M., Tomczak A., Burzyńska M., Rokosik E., Dwiecki K., Piasecka-Kwiatkowska D. (2020). Comparison of Lupinus angustifolius protein digestibility in dependence on protein, amino acids, trypsin inhibitors and polyphenolic compounds content. Int. J. Food Sci. Technol..

[40] (2009). Food and Feed Products–General Guidelines for the Determination of Nitrogen by the Kjeldahl Method.

[41] (2003). Foodstuffs–Determination of Trace Elements–Pressure Digestion.

[42] (2013). Fruit and Vegetable Juices–Determination of Sodium, Potassium, Calcium and Magnesium Content by Atomic Absorption Spectrometry (AAS).

[43] (2004). Foodstuffs–Determination of Trace Elements–Determination of Lead, Cadmium, Zinc, Copper and Iron by Atomic Absorption Spectrometry (AAS) after Microwave Digestion.

[44] Sol-Hee L., Gye-Woong K., Juhui C., Hack-Youn K. (2018). Effect of Buckwheat (*Fagopyrum esculentum*) Powder on the Physicochemical and Sensory Properties of Emulsion-type Sausage. Korean J. Food Sci. Anim. Resour..

[45] Lee M.S., Shon K.H. (1994). Content comparison on dietary fiber and rutin of Korean buckwheat according to growing district and classification. Korean J. Soc. Food Sci..

[46] Campagnol P.C.B., Dos Santos B.A., Wagner R., Terra N.N., Pollonio M.A.R. (2013). The Effect of Soy Fiber Addition on the Quality of Fermented Sausages at Low-Fat Content. J. Food Qual..

[47] Fang Z., Lin P., Ha M., Warner R.D. (2019). Effects of incorporation of sugarcane fibre on the physicochemical and sensory properties of chicken sausage. Int. J. Food Sci. Technol..

[48] Ferjančič B., Kugler S., Korošec M., Polak T., Bertoncelj J. (2021). Development of low-fat chicken bologna sausages enriched with inulin, oat fibre or psyllium. Int. J. Food Sci. Technol..

[49] Kim C.J., Kim H.W., Hwang K.E., Song D.H., Ham Y.K., Choi J.H., Kim Y.B., Choi Y.S. (2016). Effects of dietary fiber extracted from pumpkin (Cucurbita maxima duch.) on the physic-chemical and sensory characteristics of reduced-fat frankfurters. Korean J. Food Sci. Anim. Resour..

[50] Yadav S., Pathera A.K., Islam R.U., Malik A.K., Sharma D.P. (2018). Effect of wheat bran and dried carrot pomace addition on quality characteristics of chicken sausage. Asian-Australas. J. Anim. Sci..

[51] Park K.S., Choi Y.S., Kim H.Y., Kim H.W., Song D.H., Hwang K.E., Choi S.G., Kim C.J. (2012). Quality characteristics of chicken emulsion sausages with different levels of makgeolli lees fiber. Korean J. Food Sci. Anim. Resour..

[52] Bejosano F.P., Corke H. (1998). Amaranthus and buckwheat protein concentrate effects on an emulsion-type meat product. Meat Sci..

[53] Salejda A.M., Janiewicz U., Korzeniowska M., Kolniak-Ostek J., Krasnowska G. (2016). Effect of walnut green husk addition on some quality properties of cooked sausages. LWT-Food Sci. Technol..

[54] Kim H.W., Hwang K.E., Song D.H., Kim Y.J., Ham Y.K., Jeong T.J., Choi Y.S., Kim C.J. (2016). Germinated barley as a functional ingredient in chicken sausages: Effect on physicochemical and technological properties at different levels. J. Food Sci. Technol..

[55] Shin J.H., Kang M.J., Kim R.J., Sung N.J. (2011). The Quality Characteristics of Sausage with Added Black Garlic Extracts. Korean J. Food Cook. Sci..

[56] Seo J.K., Parvin R., Yim D.G., Zahid M.A., Yang H.S. (2019). Effects on quality properties of cooked pork sausages with *Caesalpinia sappan* L. extract during cold storage. J. Food Sci. Technol..

[57] Jin S.K., Choi J.S., Lee S.J., Hur S.J. (2015). Effect of *Coptis chinensis* Franch Addition on the Quality Characteristics of Sausages During Cold Storage. Food Bioprocess. Technol..

[58] Biel W., Maciorowski R. (2013). Evaluation of chemical composition and nutritional quality of buckwheat groat, bran and hull (*Fagopyrum esculentum* Möench L.). Italian J. Food Sci..

[59] Noriham A., Ariffaizuddin R., Noorlaila A. Effects of the Addition of Okara Flour on the Proximate and Amino Acid Compositions of Beef Sausage. Proceedings of the 2nd International Conference on Food Quality, Safety and Security.

[60] Jin S.K., Park J.H. (2013). Effect of the Addition of Schisandra chinensis Powder on the Physico-chemical Characteristics of Sausage. Asian-Australas. J. Anim. Sci..

[61] Romano A., Giosafatto C.V., Masi P., Mariniello L. (2015). Impact of dehulling on the physico-chemical properties and in vitro protein digestion of common beans (*Phaseolus vulgaris* L.). Food Funct..

[62] Gilani G.S., Xiao C.W., Cockell K.A. (2012). Impact of Antinutritional Factors in Food Proteins on the Digestibility of Protein and the Bioavailability of Amino Acids and on Protein Quality. Br. J. Nutr..

[63] Hur S.J., Lim B.O., Park G.B., Joo S.T. (2009). Effects of Various Fiber Additions on Lipid Digestion during In Vitro Digestion of Beef Patties. J. Food Sci..

[64] Torres J.D., Dueik V., Carré D., Bouchon P. (2019). Effect of the Addition of Soluble Dietary Fiber and Green Tea Polyphenols on Acrylamide Formation and In Vitro Starch Digestibility in Baked Starchy Matrices. Molecules.

[65] Lin Y., Chen K., Tu D., Yu X., Dai Z., Shen Q. (2019). Characterization of dietary fiber from wheat bran (*Triticum aestivum* L.) and its effect on the digestion of surimi protein. LWT-Food Sci. Technol..

[66] Ikeda S., Yamashita Y. (1994). Buckwheatasa dietary source of zinc, copper and manganese. Fagopyrum.

[67] Zając M., Guzik P., Kulawik P., Tkaczewska J., Florkiewicz A., Migdał W. (2019). The quality of pork loaves with the addition of hemp seeds, de-hulled hemp seeds, hemp protein and hemp flour. LWT.

[68] Kim H.W., Setyabrata D., Lee Y., Jones O.G., Kim Y.H.B. (2017). Effect of House Cricket (*Acheta domesticus*) Flour Addition on Physicochemical and Textural Properties of Meat Emulsion Under Various Formulations. J. Food Sci..

[69] Wahlsten A. (2019). Phenolic Compounds in Fagopyrum sp. Grains (buckwheat): Profile, Bioactivity and Effect of Processing.

[70] Dietrych-Szóstak D. Flavonoids in hulls of different varieties of buckwheat and their antioxidant activity. Proceedings of the 9th International Symposium on Buckwheat.

[71] Nikfarjam B.A., Hajiali F., Adineh M., Nassiri-Asl M. (2017). Anti-inflammatory Effects of Quercetin and Vitexin on Activated Human Peripheral Blood Neutrophils: The effects of quercetin and vitexin on human neutrophils. J. Pharmacopunct..

[72] Guimarães C.C., Oliveira D.D., Valdevite M., Fachin Saltoratto A.L., Vaz Pereira S.I., de Castro França S., Soares Pereira A.M., Pereira P.S. (2015). The glycosylated flavonoids vitexin, isovitexin, and quercetrin isolated from Serjania erecta Radlk (Sapindaceae) leaves protect PC12 cells against amyloid-β25-35 peptide-induced toxicity. Food Chem. Toxicol..

